# Nurses’ practice and educational needs in oral care for postoperative patients with oral cancer in ICUs: a multicenter cross-sectional study

**DOI:** 10.1186/s12903-022-02426-5

**Published:** 2022-09-07

**Authors:** XiaoJing Wei, MengJuan Jing, XianXian Zhang, ChunPeng Li, LiMing Li

**Affiliations:** 1 Department of Critical Care Medicine, Henan Provincial People’s Hospital, Zhengzhou University People’s Hospital, Henan Provincial Key Medicine Laboratory of Nursing, Zhengzhou, Henan China; 2 Department of Infectious Medicine, Henan Provincial People’s Hospital, Zhengzhou University People’s Hospital, Henan Provincial Key Medicine Laboratory of Nursing, Zhengzhou, Henan China; 3 Department of Nursing, Henan Provincial People’s Hospital, Zhengzhou University People’s Hospital, Henan Provincial Key Medicine Laboratory of Nursing, Zhengzhou, Henan China

**Keywords:** Nurses, Oral care, Mouth neoplasms, Practice, Primary health care, Educational needs

## Abstract

**Background:**

Surgical incision, endotracheal intubation, structural changes in the oral cavity, and other factors lead to a divergence in oral care between patients after oral surgery and ordinary inpatients. High-quality oral care can reduce the incidence of incision infection and ventilator-associated pneumonia. However, there is a lack of guidelines or expert consensus on oral care after oral cancer surgery. Therefore, the aim of this study was to assess the practicing situation of nurses in the intensive care unit (ICU) for postoperative patients with oral cancer and their need for training.

**Methods:**

A multicenter cross-sectional study design was conducted in 19 ICUs of 11 tertiary hospitals from Henan province in China. Data were collected from 173 nurses and 19 head nurses online using a structured questionnaire. Mann–Whitney U and Kruskal–Wallis H tests were performed to analyze the data using SPSS (Version 25.0).

**Results:**

Seven ICUs (36.8%) developed evaluation regulations for the oral care of postoperative patients with oral cancer, and eight ICUs (42.1%) described the operating standards. A total of 173 nurses completed the questionnaire, and the median score was 75 (68, 78). Almost all of the examined nurses (91.2%) assessed patients’ oral hygiene at a fixed time, while in 52.0% and 28.3% of nurses, the first oral care and frequency of oral care after surgery was determined based on the individual patient’s situation. More than half of the nurses (55.5%) spent approximately 5–10 min conducting oral care for patients. Physiological saline solution (82.7%), swabbing (91.9%), and oral care package with cotton ball (86.1%) were the most popular oral care mouthwash, method, and tool, respectively. Nurses sought help from senior nurses (87.3%) and doctors (83.8%), mostly to solve difficulties of oral care. Moreover, 76.9% of the nurses believed that the lack of knowledge and skills surrounding oral care was the main barrier for nurses to implement oral care. The majority of participants (69.4%) had never received continuing education or training in oral care for postoperative patients with oral cancer, and almost all (98.8%) of the respondents stated their preference to receive training in standardized oral care skills. Indications and contraindications (84.4%), tools (81.5%), and mouthwash (80.9%) of oral care were the items that the respondents were most eager to learn about. Approximately three quarters of nurses preferred scenario simulation practice as the training method.

**Conclusion:**

Although the participants had high oral care scores for postoperative patients with oral cancer, there was great diversity in the practice. The lack of oral care knowledge was deemed the main barrier in delivering quality oral care, and the educational need was stated by almost all participants. We suggest that a standard protocol or clinical practice guidelines for oral care for postoperative patients with oral cancer should be developed, and nurses should be educated to equip them with professional knowledge and skills.

## Background

Oral cancer is one of the most common malignancies in the head and neck, with a steadily increasing incidence and mortality worldwide [1–3]. Tobacco, alcohol, and human papillomavirus infections are major risk factors for patients with oral cancer [4]. Furthermore, chewing betel nuts and eating hot foods and spicy fried foods are common in some areas of China, which cause long-term adverse oral irritation and increase the incidence of oral cancer [5, 6]. A study reported that in China, as an area with a high incidence of oral cancer, the crude incidence rate of oral cancers was projected to increase from 2.26 to 3.21 per 100,000 person-years in the next 20 years [4]. According to the guidelines of the National Comprehensive Cancer Network, surgery is considered the standard treatment for oral cancer [7]. However, surgery is traumatic and time-consuming, causes heavy bleeding, and is accompanied by oral functional impairments [8]. Moreover, radical surgery for oral cancer often produces various tissue defects in the oral cavity. Patients who undergo large flap reconstruction are prone to serious complications, including swallowing dysfunction, respiratory tract stenosis, ventilation dysfunction, and even suffocation [9, 10], and are often admitted to the intensive care unit (ICU) for treatment in the early postoperative period [11].

Various microorganisms have been detected on the oral tumor surface, among which, anaerobic bacteria are dominant [12]. Postoperative patients are usually unable to eat orally because of wounds and dysphagia. Nasal administration is an important means to facilitate patients’ nutritional intake; however, this leads to a lack of food stimulation to the mouth, reduces normal oral secretions, and can easily induce bacterial reproduction. These factors not only cause incision infection but also result in ventilator-associated pneumonia (VAP) [13, 14]. Oral care, especially for patients with special oral conditions, is a basic nursing practice to maintain the comfort and dignity of patients [15]. Effective oral care can improve patients’ comfort and reduce the infection rate of the wounds and lung after oral cancer surgery. Studies have shown that proper oral care is of great significance to reduce the number of oral pathogens and postoperative incision infection in patients with oral cancer, and serves to improve patient prognosis, shorten the length of hospital stay, and alleviate the family economic burden [16–19].

The oral cleaning ability of patients with oral cancer after surgery is limited, and their oral characteristics are different from those of healthy people, leading to higher oral care requirements for ICU nurses. Due to the patient’s limited mouth opening, nurses often fail to evaluate the oral cavity before inserting oral care tools into the mouth. Moreover, nurses are often concerned that touching the patient’s oral wound will cause pain and nausea during oral care [20]. Taken together, all of these factors reduce the patient’s cooperation and increase the difficulties of oral care.

At present, the practical guidelines used to guide oral care for patients are mostly universal. However, the Chinese Nursing Society has also issued standards of oral care for mechanically ventilated adult patients with orotracheal intubation, which provides a basis for nurses in clinical practice. The British Association of Critical Care Nurses provides an evidence-based consensus on oral care for critically ill patients [21]. Nonetheless, there remain limited guidelines for oral care for postoperative patients with oral cancer, which leads to a diversity of oral care methods and implementation provided by nurses. Geyun et al. [22] showed that, compared to the traditional method of wiping with cotton balls, flushing the oral cavity was more conducive to the oral cleanliness and comfort of postoperative patients with oral cancer. This conclusion was confirmed by Yang et al. [8], who proposed that combining flushing and wiping might have more advantages. In terms of mouthwash, Jinyu Gargle, a traditional Chinese medicine preparation, improves the oral cleanliness of patients with oral cancer after surgery compared to normal saline [23]. Given the lack of evidence on the oral care methods for patients with oral cancer, the methods are diverse and still in the exploratory stage, which ultimately reduces patients’ comfort and the effectiveness of oral care [20].

Additionally, investigations into the practice of postoperative oral care for patients with oral cancer are currently insufficient. Therefore, in the current study, we investigated the current practice and educational needs of oral care for postoperative patients with oral cancer in ICUs at Chinese tertiary hospitals. The aim of this study was to provide a basis for future research to develop standardized guidance and training for nurses to provide oral care for patients with oral cancer after surgery.

## Methods

### Ethical considerations

The study was approved by The Ethics Committee of Henan Provincial People’s Hospital. Individuals were informed of the purpose of this study and the instructions of the questionnaire. Only those who agreed to participate in the survey and gave informed consent had access to the questionnaire.

### Study design and setting

A multicenter cross-sectional design was employed. The online survey was conveyed to ICU nurses enrolled in 19 ICUs from 11 tertiary hospitals conducting oral cancer surgery in Henan province, China.

### Study participants

The study was conducted from September to December 2020. The cluster sampling method was adopted, and the nurses from 19 ICUs were surveyed, excluding those who were on leave. All of the participants were registered nurses and had worked in the ICU for > 12 months. A total of 1703 nurses met the inclusion criterion and participated in the survey. To avoid recall bias, we used an adaptive question: “Have you conducted oral care for postoperative patients with oral cancer at least six times in the past month?” If the respondent answered “No”, the rest of the questions were not required to be answered and the questionnaire was submitted directly. If the respondent answered “Yes”, the respondent continued to complete all of the questionnaires. One hundred and seventy-three nurses completed the survey.

### Data collection tools

The questionnaire was designed by the study researchers after reviewing the literature [24–27] and consulting oral nursing experts. The questionnaire was reviewed and revised by five critical care experts and oral care experts who were familiar with this field. The questionnaire was divided into three parts.

#### Part I: general information

The first part (five questions) was designed to record the participants’ general data, including sex, age, educational background, professional title, and working seniority in the ICU.

#### Part II: practice of oral care

The second part covered 21 items related to oral care practice, including two sub-questionnaires: oral care evaluation (13 items) and oral care practice (eight items). All items were scored using a four-point scoring method as follows: 1, none; 2, sometimes; 3, often; and 4, always. Two items were reversely scored. The total score of this section was taken from the sum of each item, with a higher score indicating a greater level of practice of oral care. The overall Cronbach’s Alpha of the second part of the questionnaire was 0.931, and the Cronbach’s Alpha of each dimension was 0.934 and 0.891. The content validity was measured by the content validity index (CVI); the CVI of each item was between 0.80 and 1.00 and the overall CVI was 0.952.

Nine non-scoring questions involving the frequency, methods, tools and methods of oral care were also set up.

#### Part III: educational needs for oral care

The survey tool of nurses’ educational needs for oral care consisted of four questions, including whether the nurses had been trained, their training attitude, the training content, and the training form.

The electric questionnaire included 40 questions on one page, which generally took the respondents 5–10 min to complete. Once the questionnaire was submitted, the respondents could not review or change their answers.

### Data collection procedure

The researchers input the questionnaire items into an electronic questionnaire software (https://www.wjx.cn/), which is widely used in China. Each item in the questionnaire was set as required items, and the IP address of one cell phone or one computer had only one permission. The members of the research team completed the questionnaire first to test the usability and technical functionality of the electronic questionnaire before fielding the questionnaire. LM L, the chairman of the Critical Care Professional Committee of the Henan Nursing Association, explained the purpose of this study to the directors of the nursing department of the tertiary hospitals participating in the study through the WeChat group and obtained their informed consent. Subsequently, the questionnaire was gradually distributed to the ICU nurses through the path of “nursing director—ICU head nurse—ICU nurse” via WeChat.

After the completed questionnaires were collected, MJ J and XX Z checked the data separately. None of the 173 completed questionnaires had data errors, and the system showed that the time of completing the questionnaire was within a reasonable range (≥ 300 s).

### Data analysis

The data were analyzed by SPSS v25.0 software. The frequency (percentage), mean (SD), or median (interquartile range) were used to describe the variables appropriately based on their distribution variables. Differences in practice scores evaluated as non-normally distributed data were analyzed using Mann–Whitney U and Kruskal–Wallis H tests. Chi-square tests were used to compare the differences in categorical variables between groups. A *p* value of < 0.05 was considered statistically significant.

## Results

### Participants’ characteristics and scores of practice on oral care

Nineteen head nurses from 19 ICUs of 11 tertiary hospitals reported the regulations of practice of oral care for operative patients with oral cancer. Seven ICUs (36.8%) developed evaluation regulations, and eight ICUs (42.1%) described the operating standards for oral care for postoperative patients with oral cancer. A total of 173 nurses completed questionnaires on the oral care practice and educational needs for postoperative patients with oral cancer. The participants’ mean age was 30.73 (4.29) years. Most respondents (86.7%) were from general hospitals, and 146 (84.4%) nurses were female. Except for five nurses (2.9%) with master’s degree and five nurses (2.9%) with college degree, the majority of the nurses had a bachelor’s degree (94.2%). Nearly half (49.1%) of the participants possessed the professional title of nurse-in-charge, and 50.9% were primary nurses. Based on the working seniority cut-off value of 5 years, all participants were divided into three groups as follows: < 5 years (28.3%), 6–10 years (41.1%), and > 10 years (30.6%). Only 30.6% of nurses had received training on oral care for postoperative patients with oral cancer. The median score was 75, and the scores did not differ significantly according to the participants’ characteristics (*P* > 0.05) (Table 1).

**Table 1:** Differences in the scores of oral care practice according to participants’ characteristics (n = 173)

Variables	Frequency, n (%)	Scores of practice in oral care
Type of hospital		*Z* = − 0.732, *P* = 0.464
General hospital	150 (86.7%)	74 (69, 77)
Stomatological hospital	23 (13.3%)	76 (67, 78)
Age		*H* = 5.955, *P* = 0.114
~ 25 years	21 (12.1%)	76 (67, 78)
~ 30 years	63 (36.4%)	76 (68, 78)
~ 35 years	66 (38.2%)	74 (67, 78)
> 35 years	23 (13.3%)	72 (62, 75)
Sex		*Z* = − 0.939, *P* = 0.348
Male	27 (15.6%)	75 (70, 78)
Female	146 (84.4%)	75 (67, 78)
Length of work in the ICU		*H* = 0.217, *P* = 0.897
1–5 years	49 (28.3%)	76 (68, 78)
6–10 years	71 (41.1%)	74 (67, 78)
11 years ~	53 (30.6%)	75 (67, 78)
Status of education		*H* = 0.779, *P* = 0.378
College degree	5 (2.9%)	78 (69, 78)
Bachelor’s degree	163 (94.2%)	75 (68, 78)
Master’s degree	5 (2.9%)	71 (66, 78)
Professional title		*Z* = − 0.396, *P* = 0.692
Primary nurse	88 (50.9%)	76 (65, 78)
Nurse-in-charge	85 (49.1%)	75 (69, 78)

### Implementation status of nurses’ oral care

Almost all of the nurses assessed the patients’ oral hygiene at a fixed time, among whom 28.3% assessed every 4 h and 24.9% every 2 h. There were significant differences in the time of the first oral care after surgery, with more than half of the nurses (52.0%) believing that it should depend on the patient’s situation, while 38.2% followed doctors’ orders. Moreover, 30.1% of the participants provided oral care to patients according to their oral condition, and 55.5% spent 5–10 min conducting oral care for patients. Physiological saline solution (82.7%) was the most popular oral care solution for nurses, followed by chlorhexidine (a mouthwash) (57.2%). The swabbing method (91.9%) was the most commonly used in oral care, and the traditional oral care package with cotton ball (86.1%) was the preferred oral care tool. Nurses mainly sought help from senior nurses (87.3%) and doctors (83.8%) to solve difficulties with performing oral care. Furthermore, 76.9% and 74.0% of nurses believed that the lack of knowledge and skills related to oral care and insufficient understanding of the importance of oral care were important factors hindering their ability to provide oral care for postoperative patients with oral cancer (Table 2).

**Table 2:** Percentage distribution of nurses practice on oral care (n = 173)

Items	n	%
Interval for assessing patient’s oral hygiene		
2 h	43	24.9
4 h	49	28.3
6 h	29	16.8
8 h	24	13.9
12 h	11	6.4
UC	17	9.8
First time of oral care		
6 h after operation	17	9.8
Follow the doctor’s orders	66	38.2
UC	90	52.0
Frequency of oral care		
Once a day	4	2.3
Twice a day	35	20.2
Three times a day	44	25.4
Four times a day	38	22.0
UC	52	30.1
Duration of oral care		
< 5 min	26	15.0
5–10 min	96	55.5
> 10 min	19	11.0
UC	32	18.5
Method of oral care (MCQ)		
Swabbing	159	91.9
Rinsing	65	37.6
Combination of the two	84	48.6
Oral care solution (MCQ)		
Physiological saline solution	143	82.7
Special mouthwash for oral care (e.g., chlorhexidine)	99	57.2
Hydrogen peroxide solution	55	31.8
Sodium bicarbonate solution	36	20.8
Distilled water	31	17.9
Toothpaste	3	1.7
Tools of oral care (MCQ)		
Oral care package^#^	149	86.1
Large cotton swab or gauze	121	69.9
Toothbrush with flushing and suction	76	43.9
Resources of solving the difficulties of oral care (MCQ)		
Consulting senior nurses	151	87.3
Consulting doctors	145	83.8
Communicating with nurses at the same level	112	64.7
Seeking help from guidelines/expert consensus/literature	77	44.5
Barriers of oral care (MCQ)		
Lack of knowledge and skills related to oral care	133	76.9
Insufficient perception of the importance of oral care	128	74.0
Surgical incision hinders the evaluation and operation	116	67.1
Uncooperative patients due to pain and discomfort	108	62.4
Shortage of nurse human resources	100	57.8
Lack of appropriate tools of oral care	100	57.8
Lack of standardized regulation of oral care	99	57.2
Concerns about conditions caused by oral care (e.g., wound bleeding)	78	45.1
No orders of oral care	17	9.8

*UC* Uncertain, depending on the patients’ situation, *MCQ* Multiple choice questions

^#^The oral care package is a tool for oral care that includes a tongue depressor, a disposable pad towel, cotton balls, plastic hemostatic forceps, and tweezers. The cotton balls were soaked with physiological saline or oral care solution to wipe the patient’s teeth and clean the oral cavity

### Education needs for oral care

**Fig. 1:**
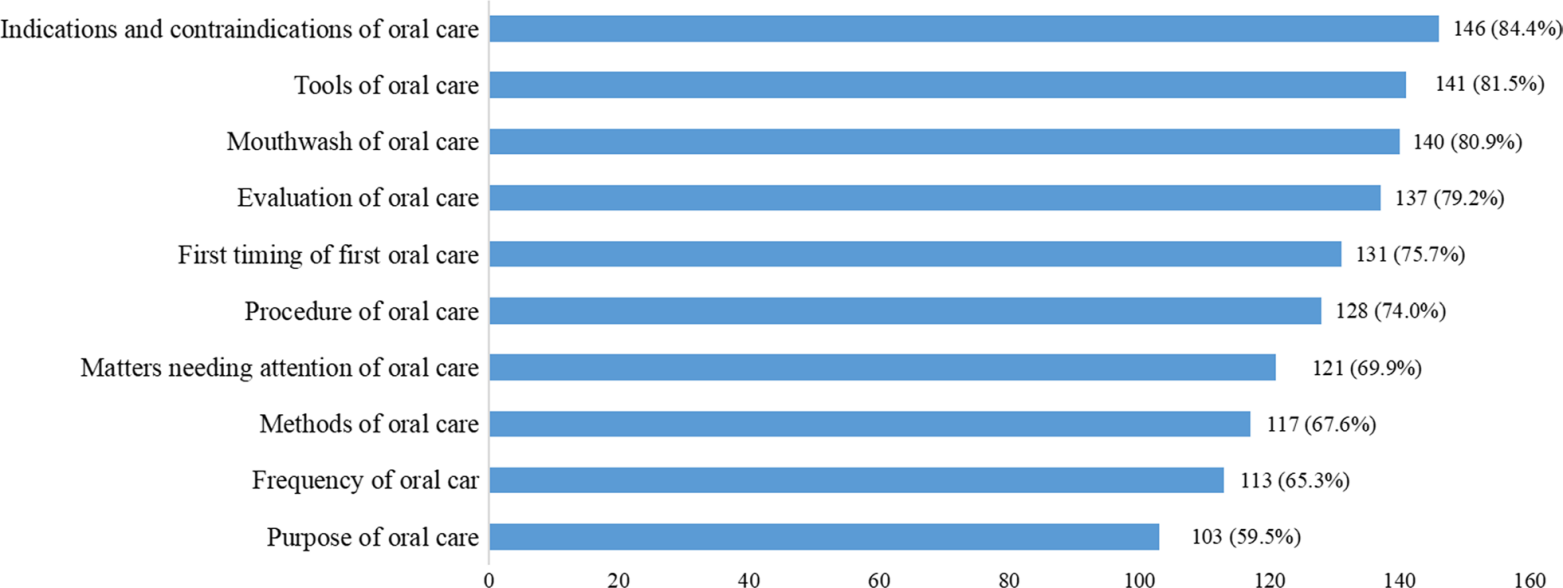
Oral care content that nurses are most eager to learn about for postoperative patients with oral cancer

**Fig. 2:**
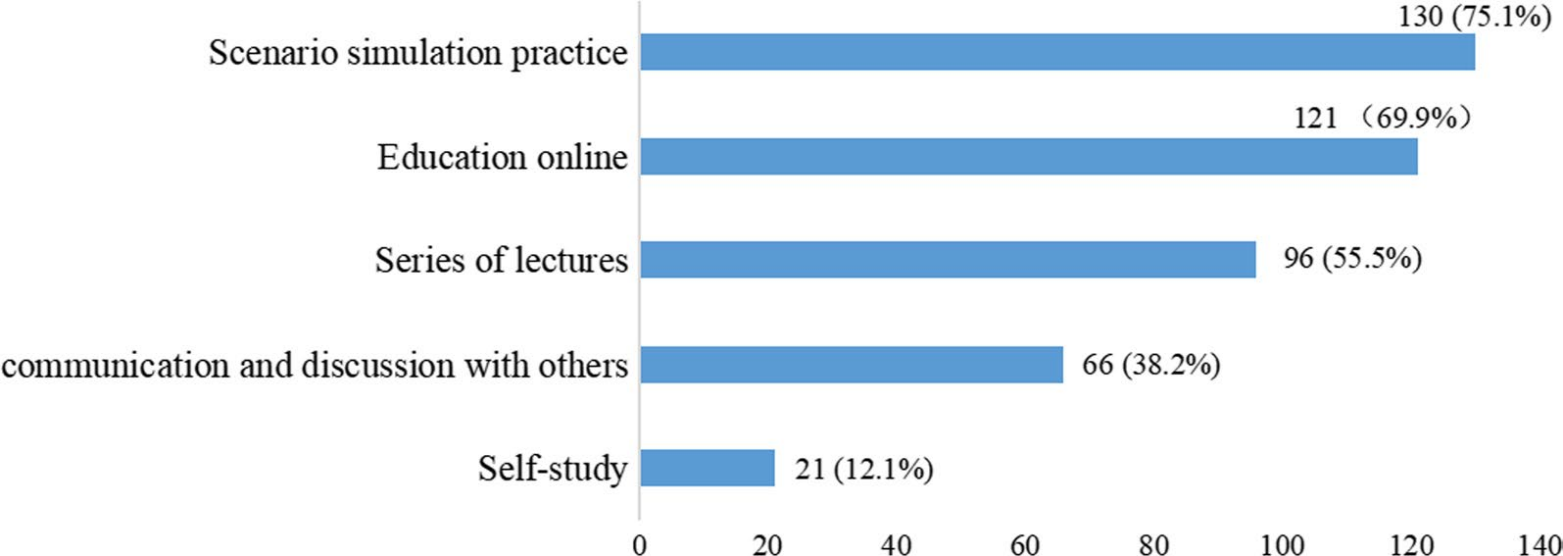
Nurses’ preferred training methods

Regarding the education in oral care, 69.4% of the participants had never received continuing education or training in oral care for postoperative patients with oral cancer, and almost all (98.8%) of the respondents had educational needs. Figure 1 shows that indications and contraindications (84.4%), tools (81.5%), and mouthwash (80.9%) relating to oral care were the items that the ICU nurses were most eager to gain further knowledge of. Approximately three quarters of the nurses preferred scenario simulation practice as the training method (Fig. 2).

## Discussion

In China’s nursing education, oral care of hospitalized inpatients has always been one of the basic skills nurses acquire. In clinical practice, particularly for critical patients with impaired self-care ability, nurses are the dominant providers of oral care. Patients with oral cancer in the perioperative period have special clinical characteristics, including dysbacteria in the oral cavity, complications caused by radiotherapy and chemotherapy, postoperative incision and flap, nasal feeding tube, and endotracheal intubation. Consequently, the role of oral care for postoperative patients with oral cancer cannot be underestimated. Beyond all doubt, training is the basis for providing high-quality oral care. However, the majority of nurses have been trained in oral care for ordinary inpatients, and little is known about the practice status of oral care for postoperative patients with oral cancer. The findings of this study revealed the nurses’ practice and educational demand for oral care of postoperative patients with oral cancer in China.

### Good compliance in the implementation of oral care for postoperative patients

The results of this study showed that the scores of oral care for postoperative patients with oral cancer were high. Indeed, most nurses chose “Always” or “Often” in evaluation and practice of oral care, and the compliance of nurses’ evaluation and practice of oral care was good. These findings were consistent with a study conducted in China that reported on the situation of evaluation and practice of ICU nurses on oral care for patients with endotracheal intubation [24]. However, the nature of the hospitals and the general demographic data of nurses were not influencing factors of the score of oral care.

### Diversity in the implementation of oral care for postoperative patients

High scores did not reflect the practicing details of oral care practice; thus, it was necessary to investigate the details of evaluation and practice in oral care. It can be seen from the results that the nurses’ implementation of oral care for the first time, including the interval of assessing patient’s oral hygiene, the frequency of oral care, and the duration of oral care, was diversified, and in most cases, depended on the patients’ situation. There were also differences in the method, mouthwash, and tools used for oral care, which may be due to the lack of guidelines and performance standards of postoperative oral care for patients with oral cancer. To explore the reasons for the diversity of oral care practice in the same department, the head nurses provided information about whether operational specifications, regulations, or workflows of oral care for postoperative patients with oral cancer were described in their wards. The survey showed that there were seven ICUs (36.8%) that had formulated evaluation regulations for the oral care of postoperative patients with oral cancer, and eight ICUs (42.1%) that had formulated operating standards, which contributed to the diversity in oral care practice.

The National Health Commission of the People’s Republic of China issued the *Clinical Nursing Practice Guide*, which defined the key points of oral care for ordinary inpatients in 2011 [28]. It was not until 2021 that the Chinese Nursing Society developed and released the first Group Standard on oral care for patients-oral care for adult mechanically ventilated patients with orotracheal intubation [29]. However, these documents lack evidence of oral care for patients with oral cancer. Although research on the oral care of perioperative patients has been adequately conducted, most of which focused on the impact of preoperative oral hygiene on the incidence of incision infection and VAP after surgery, there were few randomized controlled studies and guidelines on postoperative oral care for patients with oral cancer. In 2020, Zheng et al. [30] constructed a program of perioperative oral care for the elderly patients with oral cancer based on the content analysis of the guidelines and the Delphi method. This program provided guidance for nurses to implement postoperative oral care for patients with oral cancer. However, some important factors were unclear, such as the evaluation tools, mouthwash, and the time of the first oral care after the operation. Consequently, more RCT studies of postoperative oral care for patients with oral cancer are urgently required, and evidence-based practice guidelines should be developed to provide a reliable basis for clinical practice.

### Knowledge transformation is seldom applied in oral care for postoperative patients

Knowledge transformation helps nurses apply the best evidence-based practice clinically and improve nursing quality. Although the results showed that the latest evidence of oral care has been proven effective, ICU nurses have rarely applied it to the practice of oral care for patients with oral cancer. For example, a previous study reported that the use of a special mouthwash decreased the prevalence of halitosis and fungal infection of the oral cavity compared to the saline control group [31]. The results from a previous meta-analysis also showed that the incidence of VAP in ICU patients could be effectively decreased by chlorhexidine and povidone-iodine used as mouth gargle compared to saline [32–36]. However, this study revealed that 82.7% of participants selected saline as the main mouthwash. The percentage of nurses delivering oral care by wiping with cotton pellets was greater than 90%, while the proportion of nurses applying oral rinsing as a method of oral care was less than half, although some studies have shown that it is better to use oral rinsing [8, 22, 37–40]. This might be explained by the usage of the oral care tools, since 86.1% and 69.9% of nurses, respectively, selected cotton pellets and cotton swabs as tools to clean the mouth, while less than 50% of nurses used a toothbrush with flushing and suction. These findings confirm that information on oral health should be further disseminated, and the evidence-based nursing ability of Chinese nurses needs to be improved. The data indicated that when nurses encountered difficulties in practice, they tended to seek help from colleagues rather than the literature or practice guidelines.

### Nurses require training and education on oral care for postoperative patients

Dental hygienists play an important role in the medical service system in some countries, including USA, Japan and Netherlands, and dental hygiene has been flourishing for more than one hundred years [41]. The Working Committee of Oral Hygienist Training of Chinese Stomatological Association was established in 2018, which marks that oral hygienist will become a new profession in the field of stomatology in China [42]. However, at present, neither professional courses nor positions for oral hygienists have been offered in China [42]. Due to the lack of oral hygienists, nurses in China play the similar roles, but there are great differences in training methods and practice scope [43]. The vast majority of medical colleges and universities have not set up a specialty of oral care or oral hygiene [44]. The nurses’ training on oral care is mainly based on general nursing education, with only a small amount of knowledge about oral health [45]. This limits the improvement of nurses’ knowledge and skills on oral care. In addition, the training of stomatology specialty nurses is a continuing education program for nurses organized by the Chinese Nursing Association. However, this training program is aimed at senior nurses with clinical experience, and focuses on improving the nurses’ ability of research, teaching and management [46].

A lack of theory and clinical skills are perceived to be the primary obstacles of oral care, followed by the insufficient perception of importance of oral care for postoperative patients with oral cancer, which is consistent with the results of previous research [26]. These findings confirmed the urgent training need for oral care. Although oral care is a basic provision of nursing, many studies have shown that nurses have insufficient knowledge of oral care for different patients [47–50]. Seyed found that nurses who were qualified for oral care could improve the quality of oral health in hospitalized patients [27]. Aoki’s research [51] showed that poor nurse-dental hygienist inter-rater reliability was apparent, even if using the same assessment tool. The study also indicated that compared to professionally trained dental hygienists, nurses require further training in oral care to provide professional nursing services [51]. In this investigation, only 30.6% of nurses had received continuing education and received training on oral care for postoperative patients with oral cancer. The nurses believed that the lack of knowledge on oral care for patients with oral cancer after surgery is the primary obstacle to implementing oral care. Almost all of the nurses participating in the survey declared that they needed relevant training, which was in agreement with previous studies indicating that nurses would appreciate the opportunity to enhance and improve their knowledge and skills through training [27, 52].

We also investigated the training contents and training methods expected by nurses. The top five training contents were indications and contraindications of oral care, tools of oral care, mouthwash of oral care, evaluation of oral care, and the time of the first oral care. This result was different from a previous study, which investigated the educational needs of nurses in the field of oral health of hospitalized patients [27]. This may be due to the different patients, which also confirmed the hypothesis that nurses might have different levels of knowledge and skills of oral care for different patients.

The top two oral care education priorities for training methods were scenario simulation practice and education online. Scenario simulation practice is based on Situated Cognition Theory, which advocates taking learners as the subject to arrange learning content and connecting it with practice, so that learners can obtain knowledge and skills in practice [53]. Scenario simulation practice is suitable for the training of oral care with strong practicality. On-the-job training could also be an effective method for training oral health care skills by care professionals [54]. A curriculum of oral care for patients with oral cancer should be developed and nurses should be educated regularly to promote normative oral care.

## Limitations

As the respondents were selected from only one province in China, the generalization of the results are limited. Moreover, many respondents were excluded to improve the accuracy of the data, and the rate of questionnaire completion was only 10%, so the representativeness may have been insufficient. The other limitation of the study is that retrospect and self-reporting were used in data collection, and the participants might have overestimated their behavior. In future studies, natural observations should be preferentially applied to the evaluate the situation of nurses’ practicing.

## Conclusion

In this study, although ICU nurses had a high score in the oral care for postoperative patients with oral cancer, there were great differences in the practice among participants, including oral care methods, frequency, tools, and mouthwash. The lack of theory knowledge was considered to be the main barrier in delivering quality oral care, and the training demand was stated by almost all participants. We suggest that oral care clinical practice guidelines for postoperative patients with oral cancer should be established according to the characteristics of patients, and nurses should be educated to improve their knowledge and skills, which will directly impact the patients’ oral health and clinical outcomes.

## Data Availability

The complete data set supporting the conclusions of this article is available from the corresponding author and can be accessed upon a reasonable request.
